# A new type of protein chip to detect hepatocellular carcinoma-related autoimmune antibodies in the sera of hepatitis C virus-positive patients

**DOI:** 10.1186/1477-5956-11-33

**Published:** 2013-07-19

**Authors:** Junko Akada, Shuichi Kamei, Akane Ito, Moe Ito, Takao Kitagawa, Hiroko Furumoto, Yukari Kato, Michiko Tamesa, Motonari Takashima, Mutsunori Shirai, Hirofumi Yamano, Masaaki Oka, Yasuhiro Kuramitsu, Kazuyuki Nakamura

**Affiliations:** 1 Department of Biochemistry and Functional Proteomics, Yamaguchi University Graduate School of Medicine, 1-1-1, Minami-kogushi, Ube, Yamaguchi 755-8505, Japan; 2 Technical Research Laboratory, Toyo Kohan Company. Ltd, Kudamatsu, Yamaguchi, Japan; 3 Department of Digestive Surgery and Surgical Oncology, Yamaguchi University Graduate School of Medicine, Ube, Yamaguchi, Japan; 4 Department of Genome and Infectious Disease Control and Prevention, Yamaguchi University Graduate School of Medicine, Ube, Yamaguchi, Japan

**Keywords:** Antigen chip, Antibody profiling, Cysteine-tag, Maleimide, Hepatitis C virus, Hepatocellular carcinoma, Tumor-associated antigen, Autoantibody

## Abstract

**Background:**

We report here a new type of protein chip to detect antibodies in sera. This chip method was used to a prototype created to detect hepatocellular carcinoma (HCC) -related autoantibodies in the sera of hepatitis C virus (HCV) infected individuals.

**Results:**

Five cysteine-tagged (Cys-tag) and green fluorescent protein (GFP)-fused recombinant heat shock protein 70 (HSP70), superoxide dismutase 2 (SOD2), and peroxiredoxin 6 (PRDX6), were spotted and immobilized on maleimide-incorporated diamond-like carbon (DLC) substrates. The antibodies in diluted sera were trapped by these proteins at each spot on the chip, and visualized by a fluorescence-conjugated anti-human IgG. The total immobilized protein level of each spot was detected with anti-GFP mouse IgG and a fluorescence-conjugated secondary anti-mouse IgG. The ratio between the two fluorescence intensities was used to quantify autoantibody levels in each serum sample. Heat treatment of the chip in a solution of denaturing and reducing agents, before serum-incubation, improved autoantibody detection. We tested serum samples from healthy individuals and HCC patients using the chips. The HSP70 autoantibodies were found at high levels in sera from HCV-positive HCC patients, but not in HCV-negative sera.

**Conclusion:**

This protein chip system may have useful properties to capture a specific set of antibodies for predicting the onset of particular cancers such as HCC in HCV-infected individuals.

## Background

Hepatitis C virus (HCV) infection causes chronic hepatitis, liver cirrhosis, and hepatocellular carcinoma (HCC), which is one of the most malignant tumors and a global concern especially throughout Asia and Africa [1,2]. To avoid progression to HCC, HCV infected individuals must be monitored carefully. Biomarkers are needed for the early detection and monitoring of HCC. Alpha-fetoprotein (AFP) is a common biomarker for the diagnosis of HCC, but it is not always sufficiently sensitive to detect very early stages of HCC. Serum autoantibodies against tumor-associated antigen (TAA) offers an alternative to AFP [3,4].

Discovery of TAA has been tried mainly by three methods which differ in the method used to trap autoantibodies in sera [5]. The first is SEREX (SErological identification of antigens by Recombinant EXpression cloning), the second is SERPA (SERological Proteome Analysis) [6] or PROTEOMEX (a combination of PROTEOMics and SEREX) [7], recently, the third method, proteome-covered array chip usage for presentation of antigenic proteins has been developed [8-10]. Although several TAAs have been found using more than one of these methods, no reported TAAs show high sensitivity and specificity to detect HCC in patient sera [11-18]. As a result, combination of information from multiple autoantibodies possibly increases the HCC detection sensitivity and specificity. Such kind of diagnostic parallel detection has been attempted using several assay systems, including polystyrene microarrays, slot blot on nitrocellulose membranes, and microarrays on sol-gel derived materials [19-21].

**Figure 1:**
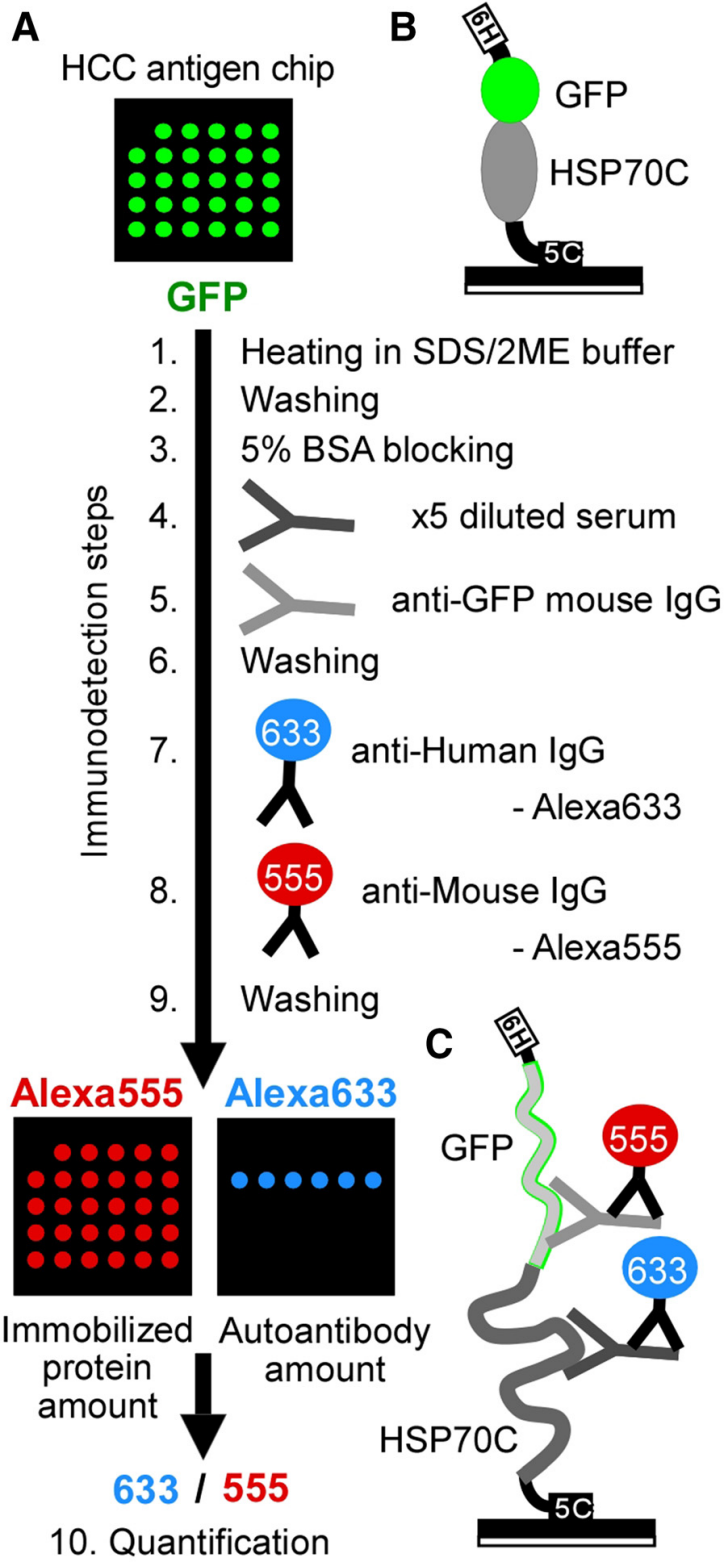
**Autoantibody detection on chip. (A)** Autoantibody detection procedures. Protein chips were immersed in SDS/2ME sample buffer and heated at 95°C for 5 min (Immunodetection step 1) and washed (step 2). The chips were blocked with BSA (step 3), incubated with diluted serum (step 4) or mouse anti-GFP monoclonal IgG (step 5), washed (step 6), incubated with AlexaFluora 633 (Alexa633)-conjugated anti-human IgG to quantify bound serum autoantibodies (step 7), incubated with AlexaFluore 555 (Alexa555)-conjugated anti-mouse IgG antibody to quantify the amount of GFP-tagged protein (step 8), and washed (step 9). The chips were examined for Alexa555 and Alexa633 fluorescence of the same spot in the same chip and Alexa633 fluorescence of each spot was normalized using Alexa555 fluorescence of the same spot (step 10).**(B-C)** Schema of predicted protein chip surface before step 1 **(B)** and after step 9 **(C)**. 6H: 6xHis-tag, 5C: 5xCys-tag.

Here, we describe a new chip-based system for the detection of multiple autoantibodies in sera from HCC patients. Model antigenic proteins to test this prototype chip system were HSP70, SOD2 and PRDX2. These three HCV-related HCC TAAs were selected from a PROTEOMEX study using the sera of Japanese HCC patients by our group, and have also been reported by others [17,18]. Using the characteristic properties of cysteine [22], cysteine-tagged recombinant antigenic proteins were immobilized on maleimide-coated diamond-like carbon (DLC)-coated silicon chips by the covalent bond between cysteine sulfhydryl and maleimide residues [23]. Theoretically, cysteine-tagged proteins on this chip were floating in surrounding solution attached to the chip substrate only at the C-terminus (Figure 1B and C), which is different from proteins bound on conventional nitrocellulose membrane via hydrophobic residues throughout the proteins. This small, hard, DLC silicon chip is stable to heat, is easy to hand for the washing process, provides sensitive detection of antibodies in low backgrounds, and is capable of high-throughput screening of sera. We constructed a procedure for the detection and quantification of antibodies bound on a cysteine-tagged protein array via maleimide residues on a DLC silicon chip. HSP70 antibodies were highly detectable in this protein chip system among HCC patient and HCV infected individual.

## Results

### Fabrication of antigenic protein array chips

Recombinant antigenic proteins of HSP70, SOD2 and PRDX6 were produced in *E. coli* BL21 strain. All proteins were constructed to contain a six-histidine (6xHis)-fused GFP-tag (26 kDa) at the N-terminus and a 5×Cys-tag at the C-terminus. A 6×His-GFP-5×Cys construct with no fusion partner (31 kDa) was used as a control. The N-terminal portion of HSP70 (HSP70N; 42 kDa) containing the ATPase domain and C-terminal portion (HSP70C; 29 kDa) containing the substrate-binding domain were expressed separately. SOD2 (22 kDa) and PRDX6 (25 kDa) were expressed as whole proteins.

**Figure 2:**
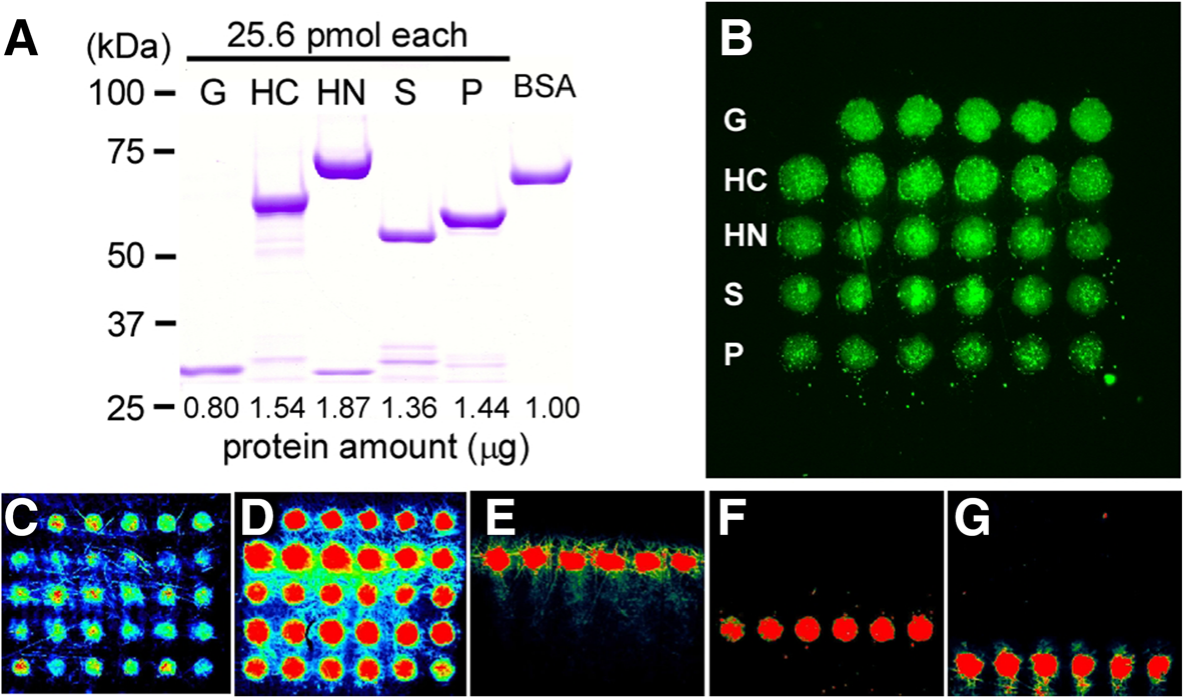
**The first-designed HCV-related HCC patient autoantigen protein chip. (A)** The five recombinant proteins confirmed by SDS-PAGE. The proteins were 6×His-GFP-5×Cys (G), 6×His-GFP-HSP70-C-terminal domain-5×Cys (HC), 6×His-GFP-HSP70-N-terminal domain-5×Cys (HN), 6× His-GFP-SOD2-5×Cys (S), and 6×His-GFP-PRDX6-5×Cys (P). **(B-G)** The first-designed protein chip and its certification. **(B)** GFP fluorescence from the protein chip as detected by fluorescence microscopy. The proteins (128 fmol per spot) were covalently cross-linked to maleimide-incorporated diamond-like carbon chip. Spots were 200 μm in diameter. Abbreviations for proteins are same as in A. **(C**-**G)** Full color fluorescence images of immobilized protein by ProscanArray. The protein chips were detected after denaturation and incubation of primary anti-His antibody **(C)**, anti-GFP **(D)**, anti-HSP70 **(E)**, anti-SOD2 **(F)**, and anti-PRDX6 **(G)**, visualized using fluorescent secondary antibodies.

The expression of recombinant GFP-tagged antigenic proteins were confirmed by SDS-PAGE after purification using Ni-affinity gel (Figure 2A). The proteins were printed on 3 × 3 mm maleimide-coated substrate chips as 10 nL, 200 μm spots containing 128 fmol protein each. The amount of antigenic proteins on each chip was verified by measuring GFP fluorescence with a fluorescent microscope (Figure 2B), and representative chips were validated by fluorescent immunodetection using specific antibodies against 6×His (Figure 2C), GFP (Figure 2D), HSP70C (Figure 2E), SOD2 (Figure 2F), or PRDX6 (Figure 2G), visualized fluorescent secondary antibodies. HSP70N (lane HN in Figure 2B) was not detected by polyclonal antibodies against HSP70 on this first-designed chip (Figure 2E), suggesting that the ATPase domain of HSP70, a functionally important conserved domain, could not be recognized by commercially available antibodies on this chip.

### Effect of denaturing antigenic proteins immobilized on chips

GFP fluorescence shows basically the amount of antigenic proteins on a chip. The protein amount in each spot is essential for quantification of autoantibodies which specifically bind to the immobilized antigenic proteins. The GFP fluorescence varied among those antigenic proteins and some of them showed aggregation on the chip (Figure 2B). This aggregation was eliminated by denaturing antigenic proteins on the chip by heating in sodium dodecyl sulfate/ 2-mercaptoethanol (SDS/2ME) solution. The antigenic proteins were then visualized by immunodetection with a monoclonal anti-GFP mouse IgG antibody followed by Alexa555-labeled anti-mouse IgG antibody (Figure 1). This indirect detection of the GFP part in fusion antigenic proteins improved the ability to detect the antigenic proteins on chips, compared to GFP fluorescence (compare Figure 2D vs. 2B), or immunodetection using anti-6×His antibody (compare Figure 2D vs. 2C). A protocol for quantitation of autoantibodies which specifically bound to denatured antigenic proteins on chips is summarized as in Figure 1. Briefly, protein chips were first denatured (as Immunodetection step 1, Figure 1), then chips were blocked with BSA (step 3), incubated with serum (step 4), followed by incubation with mouse anti-GFP monoclonal IgG (step 5). Antibodies were visualized by Alexa Fluora 633 (Alexa633)-conjugated anti-human IgG to visualize serum autoantibodies bound to antigen spots on the chip (step 7), followed by Alexa Fluora 555 (Alexa555)-conjugated anti-mouse IgG for GFP-tagged proteins (step 8). A fluorescent microscope and a microarray reader were used for detection of Alexa555 and Alexa633 fluorescence, and the Alexa633 fluorescence (antibody binding signal) was normalized on Alexa555 fluorescence (amount of chip-bound antigen protein) for each spot (step 10). The fluorescence emitted by the chips proved to be quite stable and repeated measurements could be taken.

**Figure 3:**
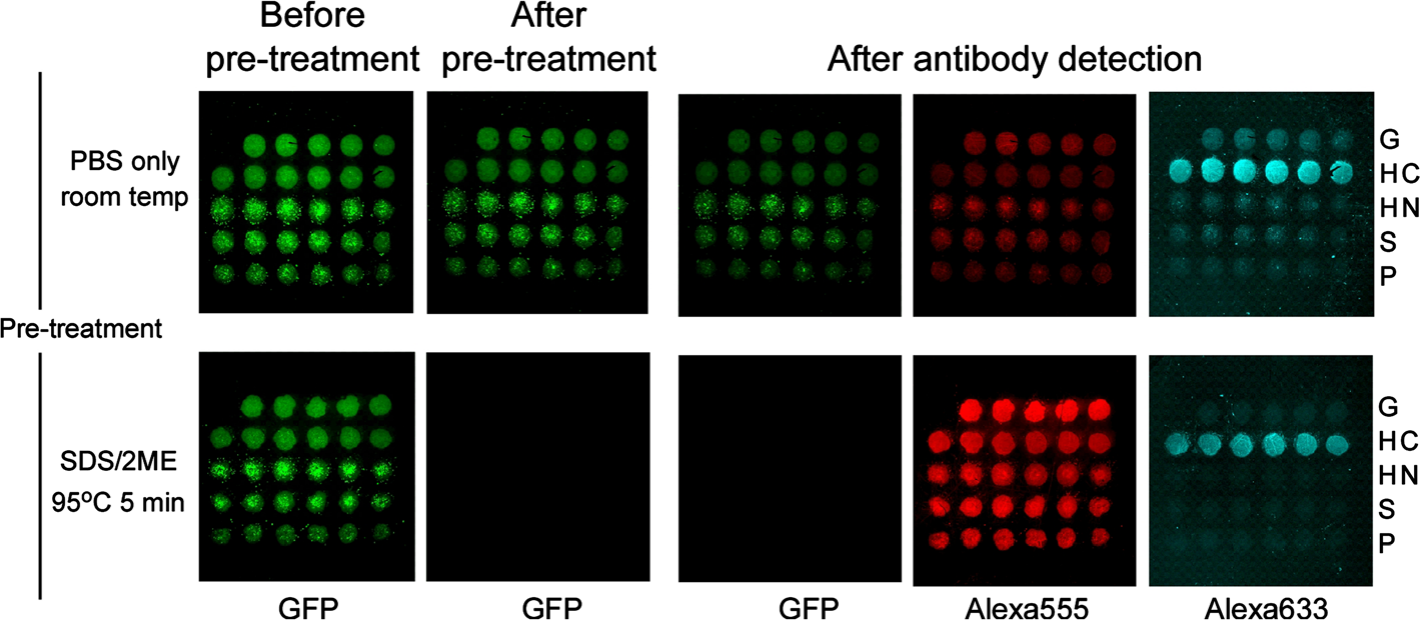
**Effect of pre-treatment for autoantibody detection on chip.** Shown are GFP fluorescence images from a non-pre-treated chip (upper row) and a SDS/2ME/heat pre-treated chip (lower row) incubated with serum no. 150. Shown are fluorescence microscopic images before (prior to step 1 in Figure 1) and after pre-treatment (after step 2) and following incubation with serum (step 10). The Alexa555 images taken at step 10 show the total amount of protein bound to the plate, and the Alexa633 images show the amount of serum autoantibodies present against the C-terminus of HSP70 (HC). Pre-treatment with SDS/2ME improved the Alexa555 signal and reduced the Alexa633 background. Abbreviations for proteins are same as Figure 2.

**Figure 4:**
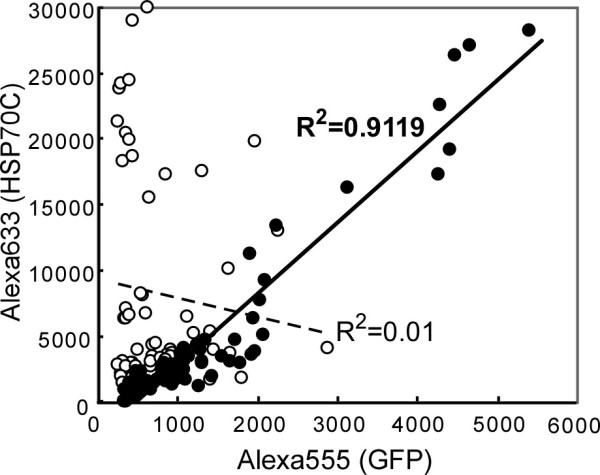
**Improvement of correlation between dual signals by SDS/2ME/heat pre-treatment.** Alexa555 fluorescence is plotted vs. Alexa633 fluorescence for each spot for chips spotted with HSP70C and pre-treated with SDS/2ME/heat (closed circles and bold line) or not pre-treated (open circles and dotted line) before incubating with serum no. 193. Each graph contains sixty spots from 10 chips for four different experiments. R^2^ values were determined by linear regression analysis. Detection of HSP70C was enhanced by pre-treatment with SDS/2ME/heat.

To check the effect of the heat-denaturing step in this protocol, GFP fluorescence was checked at each step by fluorescent microscopy. GFP fluorescence disappeared after treatment with SDS/2ME (Figure 3, lower line). In addition, Alexa555 fluorescence from the GFP tag increased for all protein spots. Alexa633 fluorescence from HSP70C remained the same, but the background was lower (Figure 3, lower line) than on non-denatured chips (Figure 3, upper line). Figure 4 shows Alexa555 fluorescence from the GFP tag and Alexa633 fluorescence from human IgG for each spot using denatured or non-denatured chip (closed or open circles, respectively). Detection of HSP70 using serum No. 193 showed that the denaturation (bold line in Figure 4) increased the linearity between Alexa555 and Alexa633 fluorescence compared to non-denatured chips (dotted line in Figure 4). It was observed that the GFP left-over fluorescence in some spots in the non-denatured chip could hardly be detected by the GFP antibody signal, and it caused lower detection in Alexa555 GFP cannels, but not Alexa633 autoantibody cannels. Carrying out denaturation as the first step mainly improved the quantitation of the GFP-tagged antigenic proteins by GFP antibody, and this in turn improved the specific quantitation of autoantibodies, which bind to the antigenic parts of the same proteins on the chips (Figures 3 and 4).

### Detection of autoantibodies specific for HCC antigenic proteins

**Figure 5:**
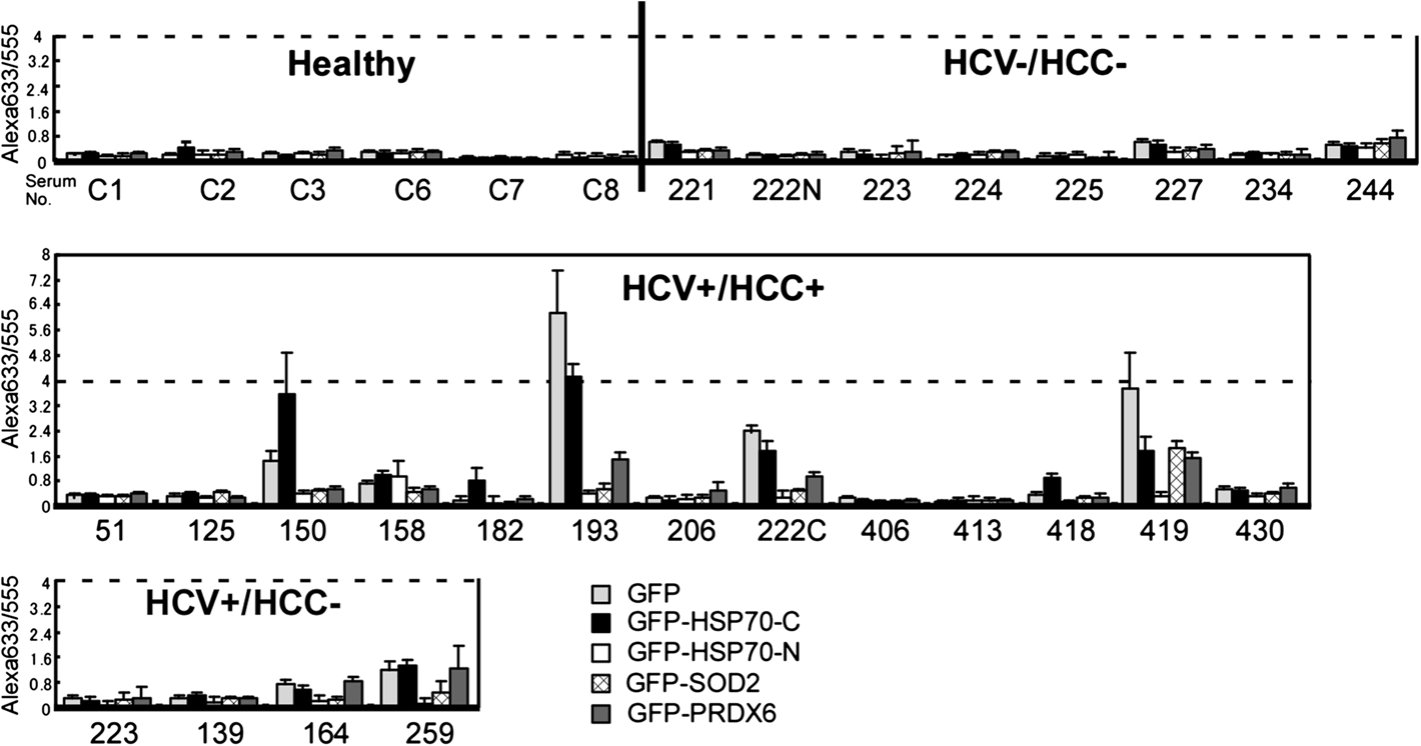
**Quantification of autoantibodies in human serum samples by the first HCC antigen protein chip.** Sera from 6 healthy individuals (Healthy), 8 HCV-/HCC- subjects, 13 HCV+/HCC+, and 4 HCV+/HCC- subjects were tested using the first designed protein chip. Each column shows the average with standard error bar of the ratio amount (Alexa633/555) of autoantibodies detected for five or six spots of GFP, GFP-HSP70-C-terminus, GFP-HSP70-N-terminus, GFP-SOD2, GFP-PRDX6 (from left to right) using one chip for each serum (showing examples in the third row of the box).

Using these protein chips and the improved detection protocol, 31 sera from four test groups were tested, including: 6 healthy individuals (“Healthy” in Figure 5), 8 subjects that were negative for both HCV and HCC, but having some other disease (HCV-/HCC-), 13 subjects that were positive for HCV and HCC (HCV+/HCC+), and 4 subjects that were positive for HCV, but negative for HCC (HCV+/HCC-). Chips incubated with sera from healthy individuals and HCV-/HCC- subjects had low antibody levels as indicated by low Alexa633 fluorescence despite strong Alexa555 fluorescence (indicating effective immobilization of GFP-tagged protein). The Alexa633/555 ratios of these negative control chips were below 0.8 (Figure 5, upper row). Chips incubated in sera from HCV+/HCC + subjects had high Alexa633 florescence for some antigen spots. The level of Alexa555 fluorescence was constant for all spots, and the Alexa633/555 ratio for the positive spots was very high (above 0.8) in four sera from HCV+/HCC + subjects (Serum No. 150, 193, 222c, 419, middle row in Figure 5), and one serum from HCV+/HCC- group (serum No. 259, bottom row in Figure 5). Thus, the protein chips could detect elevated autoantibody levels in several sera from 13 HCV+/HCC + and HCV+/HCC- subjects (Figure 5).

**Figure 6:**
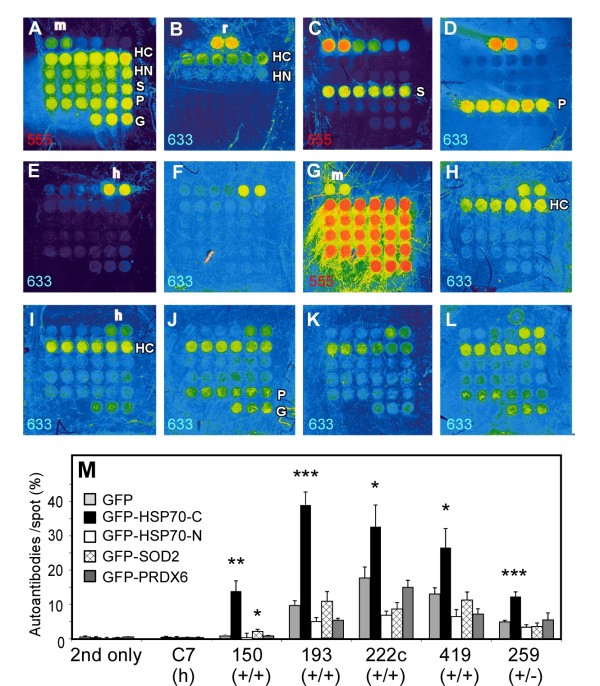
**Autoantibody detection by the second-designed protein chip, to which, 1 hr-incubation was applied for the cross-linking of antigen proteins. (A**-**L)** Full color fluorescence images of the chips by GenePi×4000B. Detected channel for Alexa555 or Alexa633 are indicated in left bottom of each figure. The abbreviations are same as for the first protein chip in Figure 2. Additionally, in the top row, two 64 fmol-spots of mouse IgG (m, detected in **A**, **C** and **G**), rabbit IgG (r, in **B** and **D**), and human IgG (h, in **E**-**F**, **H**-**L**) were immobilized for the secondary antibody detection control. **(A-D)** The protein spots certification. After denaturation step, the spots were detected by anti-GFP antibody and following anti-mouse IgG-Alexa633 secondary antibodies **(A)**, anti-HSP70 and anti-rabbit IgG-Alexa555 **(B)**, anti-SOD2 and anti-mouse IgG-Alexa633 **(C)**, anti-PRDX6 and anti-rabbit IgG-Alexa555 **(D)**. **(E**-**L)** Autoantibody detection on the chips. Autoandibody mode by ×5 diluted sera and anti-human IgG-Alexa633 **(E**-**F**, **H**-**L)**, or total protein mode by anti-GFP antibody and anti-mouse-Alexa555 (**G** only) on the same chip of **F**. Chip images of the anti-human IgG antibody only (**E**), or six serum samples tested in Figure 5, C7 (**F** and **G**), No.150 **(H)**, No. 193 **(I)**, No. 222c **(J)**, No. 419 **(K)**, and No. 259 **(L)** are shown. **(M)** Quantitative results of the chips. Autoantibodies/ spot (%) in the y-axis is showing percentage of proteins on each spot bound to autoantibody for sera, calculated after normalization of each signal mode by human IgG and mouse IgG control spots. Statistical significance of the detection from each GFP-fused recombinant antigen protein was compared with that from GFP on the same chip by the same sera, shown as *** < 0.0001, 0.0001 < ** < 0.001, 0.001 < * <0.01 by Student-*t* test. (h), (+/+), (+/−) indicates serum samples from healthy individuals, HCV+/HCC + patients, HCV+/HCC- individuals, respectively.

Unexpectedly, we found that many HCC antigen-positive patient sera reacted with GFP in Figure 5 (especially high in serum No. 193, 222c, 419). Because all antigen proteins on the chip were fused proteins with GFP, it could not be distinguished if the elevated antibody levels in these sera came from the recombinant antigen protein part or the GFP part of the fused proteins on the chip. To overcome this problem, the protein immobilization time was modified from overnight to shorter time periods with second-designed chip. It was found that the 1-hr immobilization was sufficient for the clear detection of immobilized proteins by specific antibodies (Figure 6A-D). The second chip was better in terms of the HSP70 antibody, because not only HSP70C spots were detected but also HSP70N spots were detected slightly (Figure 6B), which was not observed in the first chip (Figure 2E). We tested the second protein chip for the same HCC patient sera and controls (Figure 6E-L). Non-specific GFP detection in some tested HCC sera still remained slightly (Figure 6I, J and L three spots in the bottom row (G) on each chip). To see the significance of autoantibody detection compared with the GFP detection, we created a new quantification method.

The new method is to utilize mouse or human non-immune purified IgG protein on the same chip (m or h, respectively in Figure 6A-L). Assuming that secondary antibodies against mouse IgG spot and those against human IgG gave the same intensity, the percentage of autoantibody signal per total protein signal monitored by GFP antibody was calculated. This quantification method allowed the use of any PMT gain image of two colors for the same chip by normalization using mouse and human IgG spots (Figure 6M). Non-specific IgG binding to GFP in the same patient sera, serum No. 193, 222c, and 419, was lower than it by the previous chip (Figure 6M vs. Figure 5). In this quantification, each autoantibody signal of a serum sample was compared with its GFP signal as the statistical control. PRDX6 were not significant in any serum in Figure 6M, but HSP70C was significant among all five patient sera tested here.

**Figure 7:**
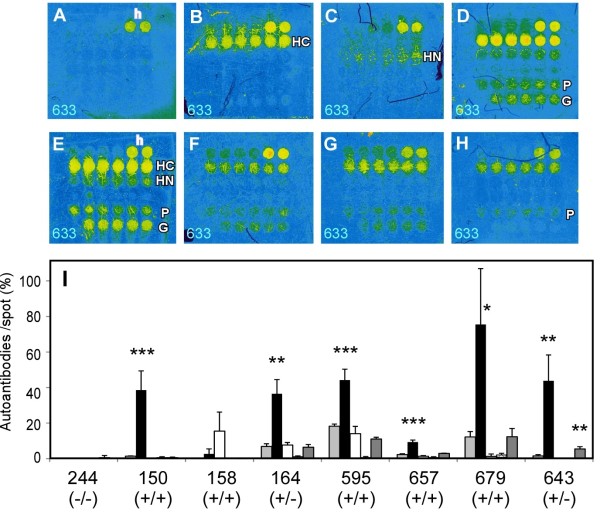
**Autoantibodies detection by the third-designed protein chip. (A**-**H)** Full color autoantibody-detected images by ×10 diluted sera and anti-human IgG-Alexa633. The representative chip images from four serum samples tested in Figure 5, No. 244 **(A)**, No.150 **(B)**, No. 158 **(C)**, No. 164 **(D)** and three new HCV+/HCC + serum samples, No. 595 **(E)**, No. 657 **(F)**, 679 **(G)**, and a HCV+/HCC- serum sample, No. 643 **(H)**, are shown. **(I)** Quantitative results of the third-designed chip. Others are shown same as Figure 6.

Furthermore, the detection procedure was modified by adjusting the fold of serum dilution (×10) and GFP antibody dilution (x5000) using the third-designed chip. Re-detection of four tested sera in Figure 5 is shown in Figure 7A-D. Negative sera in Figure 5 (serum No. 244 of HCV-/HCC- patient having some other disease) showed the same negative detection (Figure 7A), serum No. 150 and 164 increased the HSP70C detection (Figure 7B and 7D), and No. 158 was slightly positive for HSP70N (Figure 7C). Four newly tested representative HSP70C positive sera, three HCV/HCC (+/+) sera and an HCV/HCC (+/−) serum, are shown in Figure 7E-H. Quantitative analysis of autoantibodies on the third chip showed increased detection of autoantibodies and statistical significance compared with GFP non-specific detection (Figure 7I). SOD2 detection was not significant in the third chip (No. 150 in Figures 6M and 7I). PRDX6 detection was not significant except for one serum (serum 643, Figure 7H and I). It became apparent that HSP70C auto-antibody was highly significant among HCV-positive individual and/or HCC patient sera tested.

## Discussion

Autoantibodies in HCV-HCC patient sera were detected using four kinds of recombinant GFP-tagged antigenic proteins, HSP70C, HSP70N, SOD2 and PRDX6, which were immobilized by coupling of the cysteine tag at their C-terminus with maleimide groups on DLC chips. Autoantibodies specific for HSP70, SOD2, and PRDX6 in sera could be quantified by determination of intensity ratios for multicolor fluorescence from autoantibodies and antigenic proteins on chips. This method allows the detection of differences in relative levels of the four HCC autoantibodies in each serum sample. It was found that the HSP70 C-terminal domain is highly detectable by IgG in HCV infected HCC patient sera, and HCV positive sera, but not HCV negative/HCC negative sera.

This study showed that the denaturation of antigenic proteins improved the detection by increasing the availability of antigenic determinants on the immobilized proteins, especially in the GFP section of the fusion protein. The reproducible detection of GFP in GFP-HSP70 fusion protein spots, enabled the increased specificity of HSP70 detection. This method is expected to be essential to set up of diagnostic systems using multiple autoantibodies among many sera samples. Exposure of antigenic determinants along the whole length of the test antigen molecules may give benefits to increase the specific antibody detection sensitivity in this type of analysis. The authors also speculate that the 2-ME in the pre-treatment solution blocked the background of maleimide residues remaining on the chip surface and increased detection specificity. These methods were possible because of the capacity of DLC chips to undergo heat pre-treatment and also stably, covalently bind antigens [23].

It was found that many HCC antigen-positive patient sera reacted with GFP. Such GFP-reacting sera reacted with some GFP-fused antigens but not all. All recombinant antigen proteins used in this study were fused with GFP, so if non-specific GFP binding occurred, the Alexa633 and Alexa555 fluorescence intensities from all the spots should have been proportional. There were in fact a few such cases, and these were not included in the analysis (described in Methods). More importantly, healthy and HCV-/HCC- sera never reacted with GFP. The non-specific binding to GFP in some HCV+/HCC + and HCV+/HCC- sera was reduced through the modification of both the protein-immobilization process and detection process in this manuscript. The remaining reactivity of autoanitbodies in some HCC patient sera needs to be studied further.

## Conclusions

The protein chip described here is different from other chips for its capability of antigen denaturing on chip, and so it could be used for efficient detection of antibodies. This small chip system may save resources and allow high-throughput screening of sera.

## Methods

### Preparation of recombinant antigen proteins

Coding DNA sequences for human HSP70.1 (Gene bank No. NM_005345), SOD2 transcript variant 2 (NM_001024465), and PRDX6 (NM_004905) genes were cloned from human liver QUICK-Clone cDNA (Clontech TaKaRa-bio, Shiga, Japan) by PCR. These PCR fragments were then amplified using a primer set containing a *Xho*I restriction enzyme site at the 5′-end and a *Kpn*I restriction site at the 3′-end to generate the N-terminal half (ATPase domain, amino acids 1–381) and the C-terminal half (substrate-binding domain, amino acids 382–640) of HSP70.1, full-length matured (signal peptide-processed) SOD2, and full-length PRDX6. These DNA fragments were inserted into the *Xho*I-*Kpn*I sites of a 6×His-enhanced green fluorescent protein (GFP)-5×Cys-modified pET-14b plasmid to express the protein of interest with six tandem histidine residues and enhanced GFP at the N-terminus and five tandem cysteine residues at the C-terminus [23]. All plasmid constructs were made in *Escherichia coli* JM109 or DH5α using standard methods. PCR-amplified DNA fragments were verified by DNA sequencing.

*Escherichia coli* BL21 strains were transformed with newly constructed expression plasmids and the original 6×His-GFP-5×Cys plasmid (control). An inducible GFP clone was cultured in 200 mL of Overnight Express Medium (Novagen, Merck, Darmstadt, Germany) at 28°C with vigorous shaking. The bacterial pellets were lysed using BugBuster Protein Extract Reagent (Novagen) and Benzonase (Novagen). Recombinant proteins were batch-purified with 4 mL of HIS-Select Nickel Affinity Gel (Sigma-Aldrich, St. Louis, MO, USA) according to the manufacturer’s instructions except that they were washed with Econo-Column (Bio-Rad, Hercules, CA, USA) in phosphate-buffered saline (PBS, 6.4 mM Na_2_HPO_4_, 1.5 mM KH_2_PO_4_, 138 mM NaCl, 2.7 mM KCl, pH 7.4). The protein-bound Nickel-Gel beads were stored in aliquots at −80°C in an equal volume of 1:1 Tris-buffered saline (TBS, 10 mM Tris, 150 mM NaCl, pH 7.5) /50% glycerol.

After thawing, recombinant proteins were eluted from the Nickel-Gel beads with 250 mM imidazole/PBS. Buffer was exchanged with PBS by repeated dilution and concentration using a Microcon YM-10 filtration column (Millipore, Billerica, MA, USA). Protein levels and quality were assessed by Lowry protein assay and SDS-PAGE.

### Production of the protein chips

Proteins were diluted to 25.6 μmol/mL (0.796-1.87 mg/mL) in PBS and prepared for immobilization as described previously [23]. Each protein solution was printed on 3 × 3 mm maleimide-coated DLC substrate (Toyo Kohan, Tokyo, Japan) as an array of six lines, each with five (GFP) or six (other proteins) 200 μm spots of 10 nL (128 fmol; 3.98-9.35 ng) using an array spotter SPBIO2000 (Hitachi Solutions, Tokyo, Japan). The first designed chip was incubated overnight, and the second and third designed chips were incubated for 1 hr in the dark in a humidified chamber at room temperature and then washed three times with PBS. Protein chips were stored in PBS at 4°C in the dark and used immediately or within 6 months. In the second design of the protein chip (Figures 6 and 7), two spots of mouse IgG, rabbit IgG, and human IgG (Sigma-Aldrich, 64 fmol each in PBS) were immobilized on the chip in addition to the other proteins from the first chip.

For certification of immobilized proteins, after the denaturation of the chips described later, specific mouse monoclonal antibody for anti-6×His (MBL, Nagoya, Japan, 1:500 diluted, Figure 2C), and anti-MnSOD (BD, Franklin Lakes, NJ, USA, 1:1000, Figure 6C), or rabbit polyclonal antibodies for anti-HSP70 (Stressgen, Victoria BC, Canada, 1:500, Figures 2E and 6B), anti-MnSOD (Abcam, Cambridge, UK, 1:500, Figure 2F), and anti-peroxiredoxin 6 (Abcam, 1:500 in Figure 2G, 1:1000 in Figure 6D) were used and washed five times with TBS-T, then incubated for 1 hr with 1:500 diluted Alexa Fluor 633 (Alexa633)-conjugated anti-mouse or Alexa555-conjugated rabbit IgG antibody (both Invitrogen Life Technologies, Carlsbad, CA, USA, depending on the first antibodies), washed with TBS-T and PBS, same as the chips incubated with human sera.

### Serum samples

Serum samples (n = 46) from patients and healthy volunteers were collected at Yamaguchi University Hospital. The HCV antibody positivity of sera was analyzed with ARCHITECT (Abbott Laboratories, Abbott Park, IL, USA) at Yamaguchi University Hospital. The experimental protocol was reviewed and approved by the Institutional Review Board of Yamaguchi University Hospital (No. H21-91-2). Sera were diluted 5- fold (the first and second chips) or 10-fold (the third chips) with blocking solution (5% BSA in TBS containing 0.1% Tween-20 and 0.02% NaN_3_).

### Autoantibody detection by the protein chip

Chip detection is summarized in Figure 1A. Antigen-bound chips were washed with PBS for 5 min, placed in a 24-well plate, and washed with 0.5 ml TBS/0.1% Tween-20 (TBS-T) for 10 min. Chips were transferred to PCR tubes containing 200 μL of SDS/2ME sample buffer (2% SDS in 50 mM Tris–HCl (pH 6.8) with 5% 2-mercaptoethanol, 10% glycerol, and 0.0012% Bromophenol Blue), heated at 95°C for 5 min, washed five times in TBS-T, and incubated for 1 h in blocking solution (TBS-T containing 5% BSA). Chips were incubated in serum diluted five- (the first and second chips) or ten-fold (the third chips) in 50 μL blocking solution in a 96-well plate for 1.5 h with gentle shaking. Chips were then incubated for 30 min with 50 μL 1:500 (the first and second chips) or 1:5000 (the third chips) diluted anti-GFP mouse monoclonal antibody (Santa Cruz Biotechnology, Santa Cruz, CA, USA) in blocking solution. Chips were washed five times with TBS-T, incubated for 30 min with 1:500 Alexa Fluor 633 (Alexa633)-conjugated anti-human IgG antibody (Invitrogen, Life Technologies), followed by 30 min adding same volume of 1:500 Alexa Fluor 555(Alexa555)-conjugated anti-mouse IgG antibody (Invitrogen). Then chips were washed five times with TBS-T and once with PBS. All chips were placed on a slide holder, and mounted with PBS and a cover slip for image analysis.

### Image analysis and quantification of the chips

Fluorescent images of the first-designed chip were observed under BZ-9000 fluorescent microscope (KEYENCE, Osaka, Japan) and quantified by a microarray reader ProScanArray (PerkinElmer, Waltham, MA, USA) at 90% laser power for both of Alexa555 and Alexa633 fluorescence using Cy3 and Cy5 detection channels, respectively. Alexa555 and Alexa633 intensities were quantified mainly under gain 50% and gain 90%, respectively. For the chips containing spots showing strong Alexa633 intensity, each spot was detected again under gain 80%, and its 90% value was calculated from the linear relationship of gain between the 80% and 90% values (y = 2.305× - 123.62). For the chips having spots presenting less than 1000 pixels of Alexa555 intensity at gain 50%, the chip spots were detected again at gain 60%, and the 50% gain values were calculated from the linear relationship of gain between the 60% and 50% values (y = 0.276× + 3.1268). Quantitative fluorescence intensity data was collected after confirming that laser power was constant using a GEO plate (PerkinElmer). Following fluorescence detection, chips were stored for 1–12 months in sterile PBS at 4°C in the dark. For the quantification from the second- and third-designed protein chip in Figures 6 and 7, fluorescent images of each spot on chip were quantified by a microarray scanner GenePix 4000B (Molecular Devices, Sunnyvale, CA, USA) at 100% laser power using wave 532 and wave 635 channel under PMT gain 300–800. The quantified data of F635 median-B635 (auto-antibody signal) and F532 median-B532 (GFP antibody signal as total protein amount) from not-saturated images of spots on the same chip, typically 1000–20000 pixels, were used for further calculation. Quantification of autoantibody binding amounts each spot for were calculated as follows. Autoantibody/ spot (%) = (intensity of autoantibody signal normalized by intensity of control human IgG spot as 1/ intensity of GFP antibody signal normalized by intensity of control mouse IgG spot as 1) × 100. It is showing percentage of autoantibody-bounded proteins in total immobilized proteins in each spot, using normalization by human and mouse IgG control spot.

A total of eight sera among the 42 sera tested in Figure 5 (1–3 sera in each serum group) showed high fluorescence intensity for both Alexa555 and Alexa633; these samples were thus excluded from the analyses, resulting in a total of 34 serum samples analyzed in Figure 5.

## Abbreviations

HCV: Hepatitis C virus; HCC: Hepatocellular carcinoma; TAA: Tumor-associated antigen; GFP: Green fluorescent protein; HSP70: Heat shock protein 70.1; SOD2: Manganese superoxide dismutase; PRDX6: Peroxiredoxin; 2ME: 2-mercaptoethanol; DLC: Diamond-like carbon.

## Competing interests

The authors declare no financial interest.

## Authors’ contributions

TK, YKa, and JA performed experiments for recombinant protein preparation. SK, AI, and HY fabricated the protein chips. MTam, MTak, and MO contributed clinical serum samples. MI and JA performed the experiments for the chip detection of autoantibodies. HF and MS contributed in chip detection. JA, YKu and KN designed the experiments, analyzed the data, and wrote the paper. All authors read and approved the final manuscript.
